# Supplementary material for: Content-rich biological network constructed by mining PubMed abstracts
Source: BMC Bioinformatics. 2004 Oct 8;5:147. doi: 10.1186/1471-2105-5-147 (PMC528731; doi:10.1186/1471-2105-5-147)
Supplement: Additional File 2 — The original results of the above study (non-essential files are deleted to keep the file size under the limit set by BMC bioinformatics). [file 1471-2105-5-147-S2.bz2 › chilibotAdditionalFile2/dip50/281ID2498083E1104/html/FOS_JUNB.html]

 


 **FOS** and **JUNB** 
  
Found 830 abstracts in PubMed, retrieved 50.  
 

 What does Google say? 
 PDF only 
| .edu only 

---

**Interactive relationship** (e.g. stimulation, inhibition, etc)

**Neutral relationship**- Serum response factor is a transcription factor, which binds to a serum response element associated with a variety of genes including immediate early genes such as c  **fos** , fosB,  **junB** , egr 1 and 2, neuronal genes such as nurr1 and nur77 and muscle genes such as actins and myosins.  Ref: 12120892 J Physiol Pharmacol, 2002
- In CL, c Jun, JunD,  **JunB**  and Fra 2 were present in DNA binding complexes and c  **Fos**  binding was not detected.  Ref: 12606371 Biol Reprod< MedlineTA>Biol Reprod, 2002

**Non-interactive relationship** (e.g. studied together, co-existance, homology, etc.)

- We observed that withdrawal of IL 7 induced increased expression of jun and  **fos**  family member genes including c jun,  **junB** , junD, c  **fos**  and fra2.  Ref: 12034357 Cell Signal, 2002
- Possible role of transcription factor AP1 in the tissue specific regulation of human papillomavirus Activator protein 1 is a dimeric protein, consisting either of homodimers between c Jun,  **JunB** , and JunD of by heterodimers with members of the  **Fos**  family by physically interacting via a leucine zipper region.  Ref: 12183893 Rev Invest Clin< MedlineTA>Rev Invest Clin, 2002
- Further EMSA analysis using antibodies to various AP 1 components revealed that  **junB**  antibodies partially depleted the increase in binding to the PRE1 seen in UK rap1 cells while antibodies to other AP 1 constituents such as c jun, c  **fos** , and ATF 1 had no effect on binding.  Ref: 12447993 J Cell Physiol, 2003
- Phosphorylated c Jun,  **JunB** , JunD, and Fra 1, but not c  **Fos** , FosB, or ATF 2, are detected in the AP 1 DNA binding complex in cells exposed to trivalent arsenicals.  Ref: 12475910 FASEB J, 2003
- Immunoblot analyses confirmed that c Jun, JunD,  **JunB** , c  **Fos** , Fra 1, Fra 2 and FosB immunoreactive proteins were present in whole cell extracts of all antral follicles and midluteal phase corpora lutea as well as granulosa cells isolated from different sized antral follicles.  Ref: 12606371 Biol Reprod< MedlineTA>Biol Reprod, 2003
- Western blotting revealed that stress for 3 hr induced significant increases in expression of the components of AP1 complex, c  **Fos** , c Jun, and Jun B  [ **JUNB** ]  proteins, in adrenals, without markedly affecting expression of  **Fos**  B, Fra 2, and Jun D proteins.  Ref: 12271465 J Neurosci Res, 2002
- Levels were highest shortly after transient DC shifts, and decreased thereafter mono exponentially with half lives of 4 5 and 58 min for c  **fos** ,  **junB**  and MKP 1 mRNAs, respectively.  Ref: 12671253 Restor Neurol Neurosci< MedlineTA>Restor Neurol Neurosci, 2002
- Nuclear extracts from granulosa cells from 1 2 or 8 10 mm antral follicles bound an AP 1 DNA consensus sequence and complexes consisted predominantly of c Jun, JunD,  **JunB** , c  **Fos** , and Fra 2.  Ref: 12606371 Biol Reprod< MedlineTA>Biol Reprod, 2002
- In a Gal4 fusion assay, Pdcd4 specifically inhibited activation of c Jun and c  **Fos**  activation domains, but did not inhibit activation of  **JunB** , JunD, Fra 1, or Fra 2.  Ref: 12802278 Oncogene, 2003
- Our studies confirm the up regulation of c jun, jun B  [ **JUNB** ] , c  **fos**  and cyclin D1 by H. pylori.  Ref: 11966535 Aliment Pharmacol Ther, 2002
- In these mice context dependent memory retrieval evoked a marked induction of c  **Fos**  and  **JunB** , but not of c Jun and JunD, in pyramidal CA1 neurons of the dorsal hippocampus.  Ref: 12882314 Genes Brain Behav, 2003
- Genes that were commonly down regulated in both PB T cells and Jurkat T cells included CDK inhibitors p16, p19, p27, proapoptotic caspases, and the transcription factors c  **fos**  and jun B  [ **JUNB** ] .  Ref: 12808112 Lab Invest, 2003
- We found that the zinc finger region of BCL 6 interacts with c Jun,  **JunB** , and JunD proteins but does not bind c  **Fos**  or Fra 2 proteins.  Ref: 12165517 J Immunol, 2002
- Mechanistically, sodium valproate and pyridoxine significantly attenuated domoic acid induced increase in levels of glutamate, increase in levels of calcium influx, decrease in levels of gamma aminobutyric acid and increase in levels of the protooncogenes c  **fos** , jun B  [ **JUNB** ]  and jun D.  Ref: 12686137 Biochim Biophys Acta, 2003
- Memory retrieval after contextual fear conditioning induces c  **Fos**  and  **JunB**  expression in CA1 hippocampus.  Ref: 12882314 Genes Brain Behav, 2003
- To elucidate the antipsychotic potential of the selective adenosine A 1 receptor agonist N 6 cyclopentyladenosine CPA, we examined herein the effects of CPA on phencyclidine PCP induced behavior and expression of the immediate early genes IEGs, arc, c  **fos**  and jun B  [ **JUNB** ] , in the discrete brain regions of rats.  Ref: 12008016 Brain Res Mol Brain Res, 2002
- Activation of ERK1 2 resulted in induction of c jun,  **junB** , and c  **fos**  expression.  Ref: 12060661 J Biol Chem, 2002
- Similarly, in the second experiment it was found that the D1R dependent induction by AMPH of  **Fos** , FosB, and  **JunB** , but not NGFI A, in the NAc was enhanced in rats exposed 1 week earlier to repeated VTA AMPH.  Ref: 12112395 Synapse, 2002
- Acute injection of morphine induces expression of the immediate early genes c  **Fos**  and  **JunB**  in several forebrain regions of the rat, in part through an N methyl D aspartate NMDA receptor dependent mechanism.  Ref: 12809699 Neuroscience< MedlineTA>Neuroscience, 2002
- Administration of progesterone beginning on Day 20 prevented the onset of labor and blocked the expected rise in c  **fos** , fosB, fra 1, fra 2, and  **junB**  expression on Day 23.  Ref: 12080023 Biol Reprod, 2002
- Using specific polyclonal antisera against c  **Fos** ,  **JunB** , c Jun and JunD, we tried to identify the candidate transcription factors of the immediate early gene family which may contribute to the molecular processes during contextual memory reconsolidation.  Ref: 12882314 Genes Brain Behav, 2003
- Supershift and immunoblotting analyses revealed participation of c  **Fos** ,  **Fos**  B, and Jun B  [ **JUNB** ]  proteins in potentiation by kainate of mitochondrial AP 1 DNA binding in cortex and hippocampus.  Ref: 11923421 J Neurosci, 2002
- The expression of c  **Fos** , c Jun and  **JunB**  proteins was determined by the Western blot analysis.  Ref: 12411110 Chin Med J Engl , 2002
- This elevated phosphorylation of c Jun correlated with enhanced DNA binding and transcriptional activation of an AP 1 complex consisting of c Jun and  **Fos**  but lacking the c Jun antagonist  **JunB** .  Ref: 12384935 Arthritis Rheum, 2002
- The expression of c  **fos** ,  **junB**  and MKP 1 mRNAs was closely related to the time interval between the last spreading depression and the end of the experiments.  Ref: 12671253 Restor Neurol Neurosci< MedlineTA>Restor Neurol Neurosci, 2002
- Supershift electrophoretic mobility shift assay indicated that  **JunB** , JunD, and c  **Fos**  components of AP 1 were particularly affected.  Ref: 12857751 J Biol ChemJ Biol Chem, 2002
- In contrast to this generally accepted view of IKKalpha s physiological functions, we demonstrate in mouse embryonic fibroblasts that, akin to IKKbeta and NEMO IKKgamma, IKKalpha is also a global regulator of tumor necrosis factor alpha and IL 1 responsive IKK signalsome dependent target genes including many known NF kappaB targets such as serum amyloid A3, C3, interleukin 6, IL 11, IL 1 receptor antagonist, vascular endothelial growth factor, Ptx3, beta microglobulin, IL 1alpha, Mcp 1 and 3, RANTES, Fas antigen, Jun B  [ **JUNB** ] , c  **Fos** , macrophage colony stimulating factor, and granulocyte macrophage colony stimulating factor.  Ref: 12221085 J Biol Chem, 2002
- c  **fos** , jun B  [ **JUNB** ] , zif268, and N10 mRNA levels and expression patterns were analyzed using quantitative in situ hybridization histochemistry following intraperitoneal administration of selective D1 and D2 family pharmacological agents SKF 82958 and eticlopride.  Ref: 12191502 Brain Res Mol Brain Res, 2002
- VIP PACAP inhibit MEKK1 activity and the subsequent phosphorylations of MEK4, JNK, and c Jun, which result in a decrease in the AP 1 binding and a marked change in the composition of AP 1 complexes from c Jun c  **Fos**  to  **JunB**  c  **Fos** .  Ref: 12054537 Biochem Biophys Res Commun, 2002
- mRNAs and proteins for c  **fos** , c jun, jun B  [ **JUNB** ] , and jun D were all up regulated in whole lung tissues and in alveolar macrophages during acute lung injury induced by IgG immune complex deposition.  Ref: 12107112 Am J Pathol, 2002
- In contrast, mice exposed and re exposed only to the context, without foot shock, did not show behavioral signs of contextual fear conditioning and exhibited significantly less expression of c  **Fos**  and  **JunB**  in CA1 neurons.  Ref: 12882314 Genes Brain Behav, 2003
- PCP increased the mRNA levels of c  **fos**  and jun B  [ **JUNB** ]  in the medial prefrontal cortex, nucleus accumbens and posterior cingulate cortex, while leaving the striatum and hippocampus unaffected.  Ref: 12008016 Brain Res Mol Brain Res, 2002
- Towards this end, we characterized the effect pharmacological disruption of the MAPK cascade has on the expression of the immediate early genes c  **Fos** ,  **JunB**  and EGR 1.  Ref: 12752379 Eur J Neurosci, 2003
- We found that there is a lack of induction of c  **Fos** , FosB, Fra 2 and  **JunB**  by acute cocaine exposure, and of DeltaFosB by repeated cocaine administration in both the NAc and CPu of D1 receptor mutant mice compared with wild type control mice.  Ref: 12354293 J Neurochem, 2002
- In situ hybridization using oligonucleotide probes was used to study the effects of intrastriatal microinjection of corticoliberin on the expression of the early genes c  **fos** , jun B  [ **JUNB** ] , c jun, and NGFIA in the rat brain.  Ref: 12617307 Neurosci Behav Physiol, 2003
- In injured animals without SD, only a short lasting response of c  **fos** ,  **junB** , c jun and MKP 1 mRNAs as well as c  **Fos**  protein was bilaterally found in the piri form cortex, and with ipsilateral dominance the dentate gyrus and hippocampal CA3 4 fields at 1 h after lesioning.  Ref: 12671253 Restor Neurol Neurosci< MedlineTA>Restor Neurol Neurosci, 2003
- Role of CREB transcription factor in c  **fos**  activation in natural killer cells.In natural killer cells, interleukin 2 differentially regulates the expression of several transcription factors, including  **JunB**  and c  **fos** .  Ref: 12432566 Eur J Immunol, 2002
- Supershift EMSA revealed that VT preferentially induced  **JunB** , JunD, phosphorylated c Jun, c  **Fos** , and Fra 2 binding activities of the AP 1 family.  Ref: 12167214 J Toxicol Environ Health A, 2002
- Expression of c  **fos** , fosB, fra 1, fra 2, and  **junB**  was low during early gestation, with a 5 to 10 fold increase on Day 23 during labor, and returned to low levels 1 day postpartum.  Ref: 12080023 Biol Reprod, 2002
- Administration of corticoliberin into the neostriatum induced the expression of mRNA encoded by the early genes c  **fos** , jun B  [ **JUNB** ] , and NGFIA in both the neostriatum itself and in its efferent structures, particularly the nucleus accumbens and various parts of the cortex.  Ref: 12617307 Neurosci Behav Physiol, 2003
- Among the early response genes analyzed, c myc,  **junB** , junD, c jun, c  **fos** , fosB, fra, as well as max, mad1 4, sin3, only c jun and fra 2 mRNAs were up regulated after 5D exposure.  Ref: 12648525 J Steroid Biochem Mol Biol, 2003
- In this work we investigated the effect of measles virus MV infection on the expression of immediate early genes  **junB** , c jun and c  **fos**  mRNA as well as AP 1 DNA binding activity in the lung epithelial like adenocarcinoma cell line A549.  Ref: 12209312 Arch Virol, 2002
- In unilaterally pregnant rats, stretch imposed by the growing fetus was found to increase the expression of c  **fos** , fosB, fra 1, fra 2, and  **junB**  only in the gravid horn on the day of labor.  Ref: 12080023 Biol Reprod, 2002
- Using in situ hybridization, the present report demonstrates that NO released by sodium nitroprusside regulates egr 1, c  **fos** , and  **junB**  immediate early gene expression in rat forebrain.  Ref: 12105084 Ann N Y Acad Sci, 2002
- In contrast, administration of the progesterone receptor antagonist RU486 on Day 19 induced preterm labor and a premature increase in mRNA levels of c  **fos** , fra 1, fra 2, and  **junB** .  Ref: 12080023 Biol Reprod, 2002
- On the other hand, expressions of proto oncogene c jun,  **junB**  and c  **fos**  were induced by TPA and Saikosaponin a during 30 min to 6 h of treatment.  Ref: 12592382 Oncogene, 2003
